# The Role of Electronic Health Records in Advancing Genomic Medicine

**DOI:** 10.1146/annurev-genom-121120-125204

**Published:** 2021-05-26

**Authors:** Jodell E. Linder, Lisa Bastarache, Jacob J. Hughey, Josh F. Peterson

**Affiliations:** 1 Vanderbilt Institute for Clinical and Translational Research, Vanderbilt University Medical Center, Nashville, Tennessee 37203, USA; 2 Department of Biomedical Informatics, Vanderbilt University Medical Center, Nashville, Tennessee 37203, USA; 3 Department of Medicine, Vanderbilt University Medical Center, Nashville, Tennessee 37203, USA

**Keywords:** translational genomics, electronic health records, GWAS, PheWAS, PheRS, phenome

## Abstract

Recent advances in genomic technology and widespread adoption of electronic health records (EHRs) have accelerated the development of genomic medicine, bringing promising research findings from genome science into clinical practice. Genomic and phenomic data, accrued across large populations through biobanks linked to EHRs, have enabled the study of genetic variation at a phenome-wide scale. Through new quantitative techniques, pleiotropy can be explored with phenome-wide association studies, the occurrence of common complex diseases can be predicted using the cumulative influence of many genetic variants (polygenic risk scores), and undiagnosed Mendelian syndromes can be identified using EHR-based phenotypic signatures (phenotype risk scores). In this review, we trace the role of EHRs from the development of genome-wide analytic techniques to translational efforts to test these new interventions to the clinic. Throughout, we describe the challenges that remain when combining EHRs with genetics to improve clinical care.

## INTRODUCTION

Genomic medicine is an emerging multidisciplinary specialty that aims to improve human health through the application of genomic research findings to clinical care (63, 119). Arguably, it is the component of precision medicine that is most salient to clinical practice, as it builds upon the decades-long field of medical genetics and leverages well-established and increasingly affordable laboratory technologies to provide clinical-grade sequencing at the point of care. Genomic medicine is distinguished from traditional genetics in that it considers the functions and interactions of all genes in the genome (42). Thus, the field expands on the clinical model of using pedigrees to inform the diagnosis and treatment of monogenic or Mendelian disease, creating a model where polygenic effects address the hereditary components of common complex diseases, enable targeted therapy, and improve understanding of the molecular basis for all disease.

One prominent example of this evolution is in the care of patients with breast cancer. For several decades, physicians have modeled genetic susceptibility to breast cancer with *BRCA1* and *BRCA2* variants to characterize women’s predisposition to breast cancer incidence and recurrence (7, 8). However, over the last 10 years, the use of genetic data has greatly expanded to include panels of somatic and germline variants or indicators of gene expression to personalize treatment. Patients with estrogen-dependent (ER+) or human epidermal growth factor receptor 2 (HER2) oncoprotein–expressing breast cancers in particular have benefited from treatment de-escalation from chemotherapy to well-tolerated targeted therapy and hormonal prophylaxis (62, 75). Precision medicine hopes to achieve similar gains across a wide spectrum of diseases. The dramatic reduction in the cost of sequencing has enabled the study of genetic variation at the population level; the rate-limiting resource is the availability of the large populations of diverse, well-phenotyped individuals that are needed to unravel the associations between complex disease and genomic variation.

**Figure 1:**
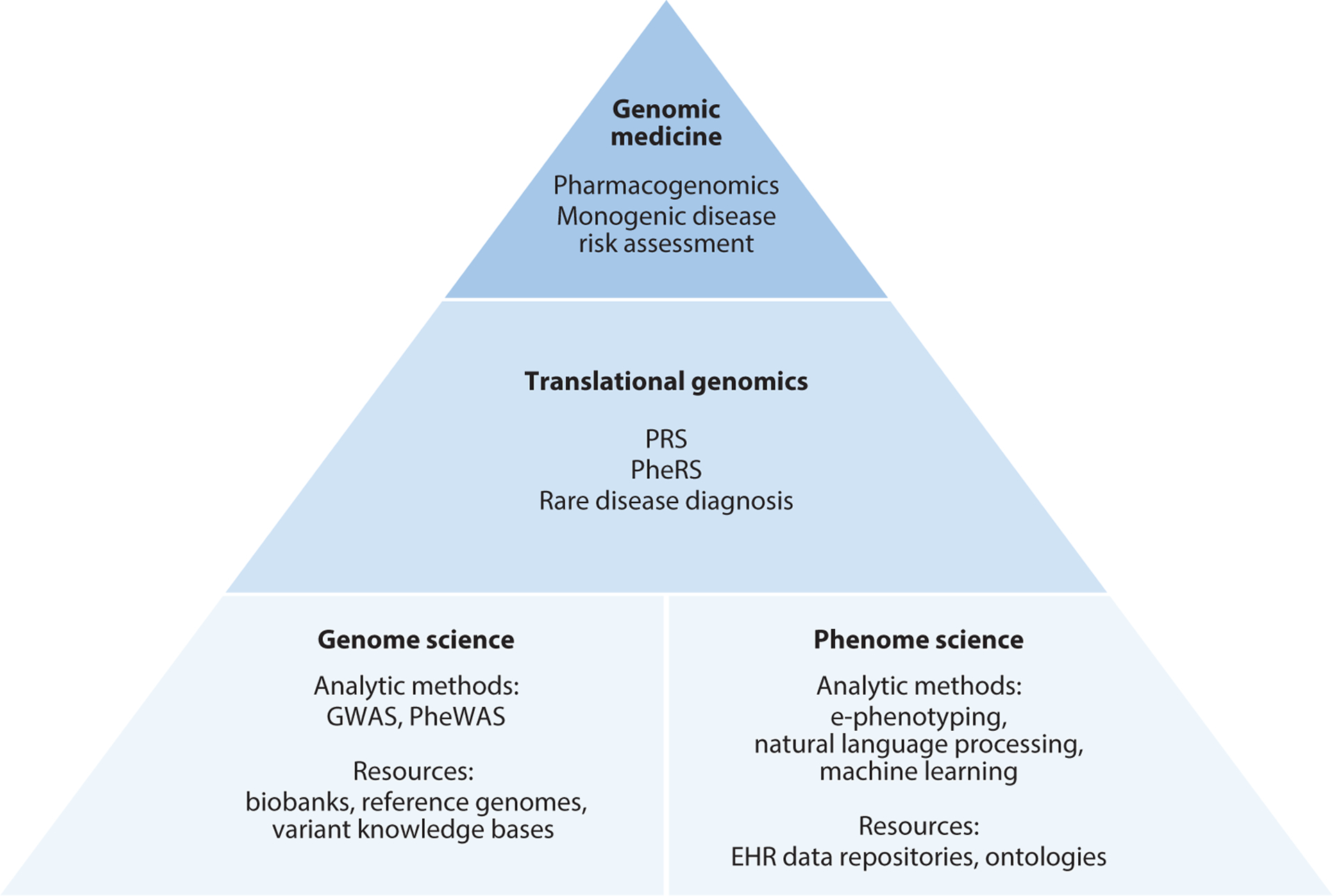
Advancing translational genomics relies on research across the genome and phenome. Progress relies both on enabling resources and on analytic methods and tools to capitalize on those resources. Discovery research utilizing new technologies built off large-scale EHR and genomic data has led to clinical translation and implementation and to eventual changes in clinical practice. Abbreviations: EHR, electronic health record; e-phenotyping, electronic phenotyping; GWAS, genome-wide association study; PheRS, phenotype risk score; PheWAS, phenome-wide association study; PRS, polygenic risk score.

It is not surprising, then, that central to the emergence of genomic medicine is the marriage of genetic data to rich sources of phenotypic data, particularly comprehensive electronic health records (EHRs) (1). In contrast to disease-specific cohorts, EHRs provide data on a complete spectrum of human disease, treatment effects, and outcomes. EHRs are foundational for phenome science, defined as the study of phenotypic characteristics across large populations (Figure 1). Both genome science and phenome science have required the development of large-scale analytic methods and resources to extract and organize vast amounts of data and draw meaningful conclusions. The development of these techniques, including phenome-wide association studies (PheWASs) (23, 24), genome-wide association studies (GWASs) (38, 43, 56, 74), and electronic phenotyping (e-phenotyping), is the subject of this review, along with the derivative translational methods of phenotype risk scores (PheRSs) and polygenic risk scores (PRSs), which are promising new interventions that may further influence clinical practice.

## UTILIZING ELECTRONIC HEALTH RECORDS TO ENABLE GENOMIC SCIENCE

EHRs, which were introduced more than 50 years ago (34, 69), did not gain substantial popularity in the United States until 2007, when federal funding from the Health Information Technology for Economic and Clinical Health (HITECH) Act (47) prompted rapid adoption. Within eight years, more than 80% of federally incentivized hospitals had adopted EHRs, and clinical data for a large majority of the US population (>95%) began accumulating (3); similar trends have occurred in other countries that have implemented national record systems.

Since EHRs automate the collection of clinical data as they are generated, they provide a unique opportunity to define disease incidence, trajectory, and outcomes across an entire health system or, in international settings, a national population. The array of data available within EHRs (Table 1) also provides a broader and potentially more nuanced representation of the phenome than is found in most clinical research cohorts. For example, findings from radiographic, laboratory, and procedural reports provide objective confirmatory evidence of disease that complements administrative codes and problem list entries, and also provide clinical details to allow disease staging and other metrics of disease severity. In addition, longitudinal EHR data enable investigators to examine how risk factors and disease are interwoven over the course of an individual’s life span. Both of these EHR features allowed investigators from a US-based integrated health system to study the impact of familial hyperlipidemia–related variants over patients’ lifetimes (2). Specifically, they were able to demonstrate the association between familial hyperlipidemia–related variants and low-density lipoprotein (LDL) cholesterol, and then the cumulative effect of elevated LDL values on the lifetime risk of ischemic heart disease. In this study, the risk for premature coronary artery disease (defined as having the disease at age 55 or younger in males and age 65 or younger in females) among familial hyperlipidemia variant carriers was particularly notable, with an odds ratio of 3.7 compared with noncarriers, reinforcing the prognostic importance of knowing one’s familial hyperlipidemia status early in life. Without the availability of decades of EHRs across a large, sequenced population, such studies are not feasible.

However, EHR advantages are balanced by common limitations of using EHR data for clinical research. The primary challenge is the completeness of patient records; some records may be fragmented across different health systems or interrupted when new EHRs are implemented or migrated to a new vendor (110). Records may be “left-censored” prior to the date the patient begins receiving care at an institution and “right-censored” at the point the patient exits the care of that institution (13). EHR-based cohorts are also not population-based samples and represent only those populations that have access to and can afford care at that institution. Both of these limitations indicate that the lack of a phenotypic signal within patient records does not always constitute strong evidence for the absence of that phenotype; sufficient detail may simply be missing from the available EHRs. Investigators have developed several strategies for mitigating these sources of bias, including the use of a “medical home” population that is likely to receive longitudinal primary care at the institution hosting the EHR (13). This strategy narrows the retrospective study cohort to those with repeated historical visits at specific clinics. A second strategy is to cross-link registry, state, or other external data sources to fill in gaps in local EHR data and/or provide corroborating signals. Overall, the limitations of EHRs are outweighed by the wealth of clinical information that is available. The ability to use these data in a high-throughput mechanism and link to genomic data is critical to the advancement and practice of genomic medicine.

Access to EHR data for research purposes requires the development of a parallel resource, the clinical data warehouse, which provides data to investigators in formats conducive to large-scale research (70). Though clinical data warehouses derived from EHRs can be costly to build and maintain, the investment can facilitate rapid translational and discovery-based research. At this time, there is no unified approach to constructing a clinical data warehouse; a recent comprehensive review found approximately 29 separate data architectures for these data repositories (38). This heterogeneity complicates the pooling of data across institutions and is part of the reason that the development of e-phenotyping algorithms requires validation at multiple institutions to demonstrate portability.

### Development of Electronic Phenotyping

As EHR data accumulated over decades, researchers began to utilize highly structured data types to represent phenotypes, or the observable characteristics of an individual resulting from the interaction of one’s genotype with the environment. The earliest e-phenotyping methods are founded on the common denominator of the administrative coding that underlies the process of billing for healthcare. In the United States, the Medicare program was instrumental in requiring diagnostic and procedural codes in machine-readable formats, which initially allowed researchers to determine causes of hospitalization in elderly populations over time (67). This schema comprises two key code sets: the International Classification of Diseases (ICD) codes and the Current Procedural Terminology (CPT) codes. Now in its 10th revision, the ICD diagnostic codes are used in the majority of disease-based phenotype algorithms developed in the last decade. As researchers began to utilize electronic code data, issues with accuracy began to arise (50, 60), and grouping and collapsing codes to increase diagnostic reliability was recommended (89). These efforts grew into early e-phenotyping (15, 57), where researchers utilized combinations of billing codes and discharge data to define cases and controls for diseases and clinical outcomes. As institutions began to organize their data and create integrated data warehouses (38), the breadth of data available for research grew beyond standard codes and administrative data to include laboratory results, medications, vital signs, medical notes, and reports (115). Table 1 lists commonly utilized data elements gathered from EHRs and the associated utility for phenome science.

In 2010, Ritchie et al. (84) developed e-phenotypes for five conditions of interest and examined genetic associations across a large biobank population; by replicating known associations and discovering new ones, the team demonstrated the utility of e-phenotyping for establishing genome–phenome associations. The need to improve phenotype fidelity prompted investigators to develop tools to extract more complex data (96). What started as more structured, rule-based algorithms moved to methods such as natural language processing (95, 120), deep data mining, machine learning (121), and artificial intelligence (51). These techniques have allowed researchers over the last decade to scan both the unstructured and structured components of EHRs. For example, a combination of natural language processing–derived disease concepts, administrative codes, and laboratory results can define a broad spectrum of ischemic heart disease risk factors; analysis of these longitudinal data using machine learning greatly increases the discrimination of cardiovascular disease predictivity (121). The portability of these techniques (93) across institutions and data systems is critical to move from research on custom cohorts and populations to large-scale, cross-institutional, translational research. The organization of EHR data into integrated research warehouses allowed for high-quality phenotypes in large cohorts, and the standardization of these data warehouses into common data models precipitated the era of large-scale data sharing.

### Transitioning from Local Data to Large-Scale Collaborations

To facilitate cross-institutional analyses, electronic health data must be standardized to minimize the bias of local institution data storage, terminology, and formats. Over the last two decades, the use of common data models has increased in many research programs, enabling researchers to develop analyses locally and rapidly implement them across external institutions. Examples include the Observational Medical Outcomes Partnership, established after the Food and Drug Administration Amendments Act of 2007 (35), which required the Food and Drug Administration to collaborate with public and private partners and access disparate data sources to increase safety data analyses (100). The Observational Medical Outcomes Partnership has grown into the Observational Health Data Sciences and Informatics program, which has more than 2,500 users from 19 countries and half a billion patient records from more than 100 different databases. The National Patient-Centered Clinical Research Network (PCORnet) (17) has demonstrated the ability to collect large quantities of strictly curated EHR data across more than 70 million people and 11 research networks and to create a coordinating center using a common data model. This rigorous structure allows for more rapid data collection at lower costs, effectively giving researchers access to a large, nationwide EHR data set. Other examples include the Shared Health Research Information Network (SHRINE), which aims to enable population-based research through large-scale data sharing and is key to bridging the gap between small discovery-based cohorts and larger translational studies, and the Informatics for Integrating Biology and the Bedside (i2b2) tool, which aims to enable precision medicine through open source data sharing, standardizations, and integration.

These large networks allow researchers to quickly respond to emerging diseases. For instance, the National COVID Cohort Collaborative was quickly set up through a partnership between the National Center for Advancing Translational Sciences and the Clinical and Translational Science Awards program in the spring of 2020 and rapidly established an infrastructure for accepting, aggregating, and providing expedited access to EHR data on coronavirus disease 2019 (COVID-19) patients to support cutting-edge research during the pandemic. The data structures, based on the i2b2, PCORnet, Observational Medical Outcomes Partnership, and TriNetX common data models acquire data twice monthly from more than 50 different institutions. Without standardization, analyses would be limited to local instances or come with high costs in effort and funds to transform analyses across data warehouses.

Common data models and large institutional data warehouses have facilitated the increase in high-throughput research over the last decade and enabled large-scale clinical research. However, it is the linkage of these data to genomics that enables precision medicine and the success of translational genomics research.

### Large-Scale Biobanking-Enabled Genomic Research

Preserving biospecimens for later research is a fundamental component of both discovery and translational studies. Biobanks can range from small, study-specific repositories to large, institution-wide efforts. The establishment of institutional biobanks in hospital settings has allowed researchers to preserve specimens collected through routine clinical care. This allows for banking of specimens already being sampled for clinical purposes, reducing the burden on patients. For example, groups such as the National Cancer Institute have developed strategies and operational procedures to maximize the creation of standardized, sustainable resources (58, 106). Biobanks with prospective enrollments offer the ability to use germline DNA for large-scale genomics. Examples like the Vanderbilt University Medical Center bank BioVU (87) enroll participants and then obtain discarded blood collected through routine care, allowing the participant to contribute to the DNA bank without requiring an additional blood draw. Large-scale genomic biobanks include the *All of Us* Research Program (4, 18), which focuses on enrolling a million participants with an emphasis on underrepresented populations; the UK Biobank (19, 76), which followed and collected data on 500,000 participants across the United Kingdom, tied these data to genomic data, and made the data available to researchers across the world; and the Electronic Medical Records and Genomics (eMERGE) network, which collected EHR and genomic data on more than 130,000 participants across the United States.

Hundreds of biobanks exist across the world (77, 101), setting the stage for advances in a variety of diseases and overall health. These specimens can be used to answer genomic, epigenomic, proteomic, and metabolomic research questions. Biobanks storing blood or extracted DNA have the potential to examine genomics across large cohorts and diverse populations. The ability to tie these biobanks to longitudinal EHRs is critical when it comes to examining large-scale genomic research. A few data points collected at time of enrollment, or a snapshot of an EHR, does not allow for large-scale data mining, longitudinal data, or the ability to assess disease outcomes. Interfacing with participants requires time, study staff effort, and participant education and can be costly, making the ability to tie genomics to on-the-shelf large-scale EHR data paramount. As reviewed by Stark et al. (101), governments around the world are making investments in genomic medicine initiatives to help bridge the gap between discovery research and translational medicine, and these initiatives aim to collect genomic data tied to clinical health records.

Several factors contribute to the ability to move from local disease-specific analysis to large-scale translational genomics work. First, the ability to effectively mine large-scale EHR data utilizing electronic methods and tools allows researchers to look across their data warehouse for associations in local patient populations over time. Second, institution-wide efforts to share data in structured formats across institutions facilitate researchers’ ability to nationally and globally share data. This paves the way for research focused on rare and common disease, increases the ability to examine conditions over diverse populations, and contributes to national and global efforts. Finally, the ability to tie these large-scale EHR data to genomics empowers investigators to take the next step in translational and precision medicine, allowing the field of genomic medicine to rapidly increase and diversify. This linkage launches myriad tools and techniques that today are leading to a new era of translational genomics research.

## FROM DISCOVERY TO CLINICAL TRANSLATION

### Genome-Wide Association Studies to Polygenic Risk Scores

**Figure 2:**
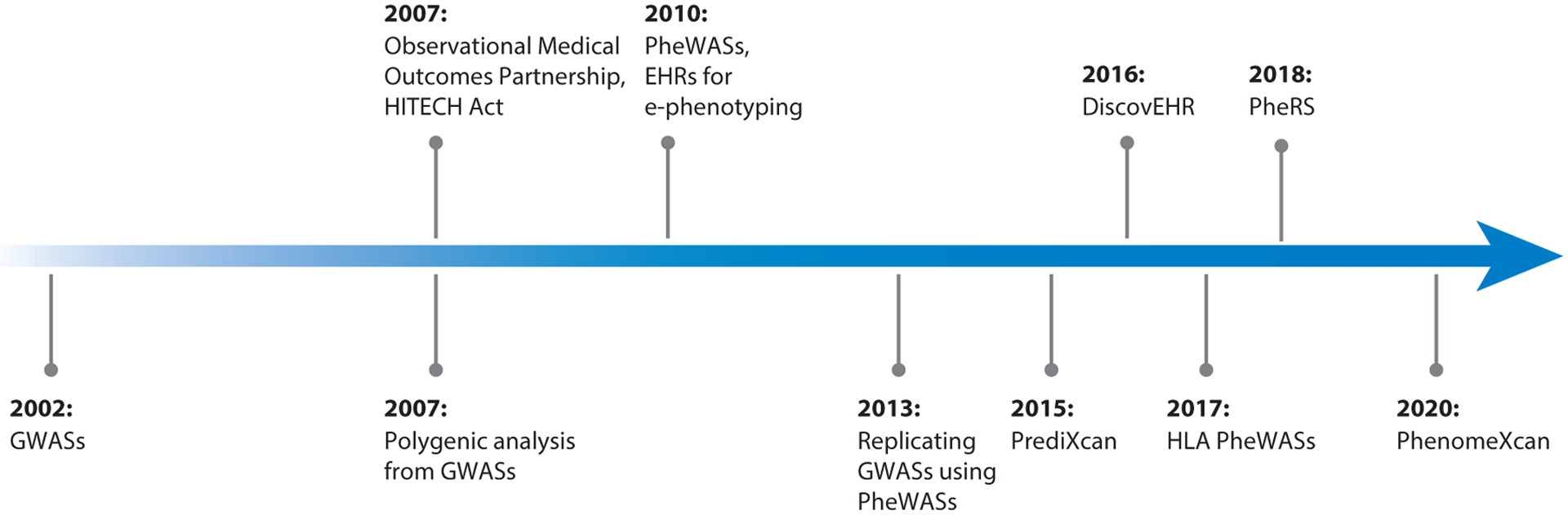
Milestones enabling translational research. EHR (*top*) and genomic data (*bottom*) technologies facilitated advancements in medical genomics, increasing the understanding of common complex diseases. Abbreviations: EHR, electronic health record; e-phenotyping, electronic phenotyping; GWAS, genome-wide association study; HITECH, Health Information Technology for Economic and Clinical Health; HLA, human leukocyte antigen; PheRS, phenotype risk score; PheWAS, phenome-wide association study.

The theory behind using large-scale genomic associations to understand common complex disease was proposed by Risch & Merikangas (83) in 1996. GWAS technology was developed in 2002 (74) to agnostically search for genetic associations with a single trait, and was implemented shortly thereafter, in 2005, to examine genome-wide associations for single-nucleotide polymorphisms (SNPs) involved in age-related macular degeneration (43, 56) (see Figure 2). The success of early GWASs incentivized researchers to find more efficient ways to study large cohorts. By 2007, both the Wellcome Trust Case Control Consortium (111) and the Framingham Heart Study (11) were publishing studies that addressed multiple phenotypes in the same cohort. Using shared controls for multiple phenotypes streamlined GWASs by reducing the number of subjects who needed to be genotyped. GWASs set the stage for heritability estimates of SNPs to be associated with common complex diseases. GWAS analyses with imputation have shown that the heritability of many common diseases can be explained by common variants with small effect sizes across the genome (104, 108). Utilization of GWASs expanded rapidly, with 4,771 publications and 214,295 associations in the GWAS Catalog (12) as of November 2020.

GWASs also allowed researchers to examine polygenic associations of diseases across the genome. The initial association studies that used GWAS data in humans to examine polygenic risk focused on risk of psychiatric disorders, cancers, and cardiovascular disease (49, 97, 116). This work paved the way for the field of PRSs. Over the last several years, PRSs have facilitated the transition from genomic discovery research using GWASs to clinical translational work, associating risk values, odds ratios, and statistical confidence with genomic associations. SNP selection and weighting (how likely it is that the SNPs are associated with the condition of interest) are used to model and validate polygenic risk (14).

PRSs allow investigators to utilize the cumulative effect of relatively common genetic variants that may contribute to common complex diseases. They have gained traction in neurological disorders like schizophrenia (65, 105) and Alzheimer’s disease (33), as well as many common complex diseases, such as colorectal cancer (103), prostate cancer (118), coronary artery disease (54, 102), atrial fibrillation, inflammatory bowel disease, type 2 diabetes (54), type 1 diabetes (94), and breast cancer (54, 66, 90). Several of these studies have demonstrated that PRS risk can be equivalent to monogenic risk (54, 66), suggesting that PRSs will also have clinical utility for predicting incident disease and tailoring preventative care. The initial work on PRSs has led to randomized controlled trials to examine their utility in clinical settings. A 2017 trial and meta-analysis of two other randomized controlled trials on statin usage for individuals with atherosclerosis risk found that those in the highest genetic risk categories derived greater relative and absolute benefit from the statins and reduction in coronary heart disease events (72) than those in other risk categories.

PRSs are an example of how the integration of large-scale genomics to examine multiple components of disease development can drive translational research. Though the evidence base for the clinical utility of PRSs is growing, and some (such as breast cancer and cardiovascular disease) have been incorporated into clinical risk scores (14, 104), several pitfalls have emerged. The performance of PRSs across males and females (44) and across ancestry groups is not always maintained, and applying them without adjustment may exacerbate health disparities in underrepresented populations (65). This is partially because the first generation of PRSs are derived from GWAS data sets that do not have sufficient numbers of non–European ancestry individuals. Recent work has moved to the generation and validation of PRSs in more diverse cohorts (5, 28, 73) and trans-ancestry modeling (26, 61) to help mitigate issues of translatability to clinical populations. The capabilities of PRS research and its ultimate clinical utility rely heavily not only on the genomic data available but also on links to the phenome. Because polygenic risk does not necessarily demonstrate the whole picture of disease development (30, 71), clinical factors, family history, and monogenic risks must also be considered. The ability to determine which individuals have conditions and traits of interest and the connection of these conditions and traits to the genomic information has taken place through advancements in biobanking, EHR mining, and data capitalization as well as the capability for large-scale data sharing over the last 10 years.

### Development of Phenome-Wide Association Studies to Conduct Large-Scale Analysis of Human Phenomes

GWASs were enabled by genotype array technology that allowed researchers to sample genetic variation across the human genome. Similarly, the adoption of EHRs enabled querying of a broad spectrum of signs, symptoms, diagnoses, and laboratory and radiographic findings across the human phenome. The breadth of phenomic data in EHRs motivated the introduction of a large-scale method representing the analytic inverse of a GWAS: a PheWAS. PheWASs scan a large set of diagnoses or other clinical findings to identify phenomic features associated with single genetic loci (24). One initial application included the exploration of genetic pleiotropy—the phenomenon where a single gene influences multiple traits (23). For example, the PheWAS technique has been used to identify potential functions for the highly polymorphic human leukocyte antigen (HLA) genes encoding major histocompatibility complexes involved in immune processes (45, 52).

Underlying the PheWAS technique is a knowledge base of diagnostic codes that can characterize a cohort on a phenome-wide scale; manually grouped administrative codes are binned to create phecodes that each represent a single disease entity. Phecode mappings (109, 117) can be found at https://phewascatalog.org (23). Currently, phecodes are defined for more than 1,800 diseases, symptoms, and clinical findings (109, 117).

PheWASs have been validated in part by replicating known genotype–phenotype associations; for example, a PheWAS exploring genetic associations with seven diverse diseases replicated four of seven previously established findings (24). Subsequently, a larger study replicated 51 of 77 associations reported in the GWAS Catalog for which there was a matching phecode (23). These studies showed that phenome-wide characterizations of EHR cohorts could be used for both validation and discovery. However, the results also showed that phecodes and large-scale analytic methods such as PheWAS trade some precision for breadth. Replicated PheWAS associations often exhibit an attenuated effect size compared with the original GWAS. While some attenuation is expected due to regression to the mean, some loss of signal may also be related to the drawbacks of scalable billing code–based phenotypes, which are subject to loss of sensitivity and specificity.

### Application of Large-Scale Phenomic Analyses

**Figure 3:**
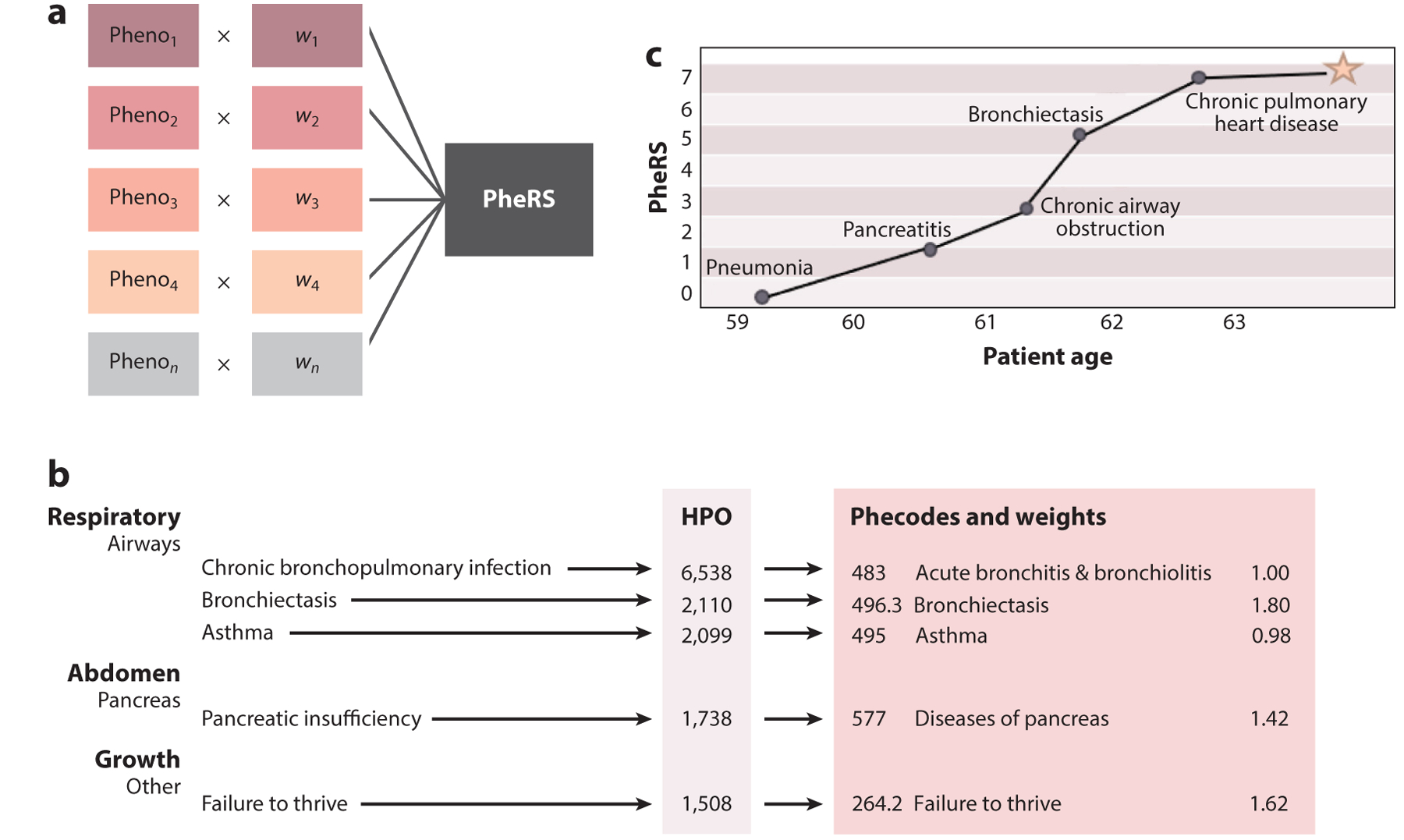
Creating a PheRS (see also the sidebar titled Creating a Phenotype Risk Score). (*a*) Summing the weights of each feature present in an EHR to calculate the PheRS. (*b*) An abbreviated version of OMIM’s clinical description for cystic fibrosis. (*c*) An example PheRS plot for a patient diagnosed with cystic fibrosis late in life. Before the diagnosis, this patient had a cystic fibrosis PheRS in the 99th percentile. Abbreviations: EHR, electronic health record; HPO, Human Phenotype Ontology; OMIM, Online Mendelian Inheritance in Man; PheRS, phenotype risk score.

The development of PheWASs has inspired more recent work to leverage EHRs to identify genetic syndromes that have a complex phenotypic expression. The PheRS method was initially created to study the impact of rare genetic variants and Mendelian disease (see the sidebar titled Creating a Phenotype Risk Score along with Figure 3). PheRSs use clinical descriptions taken from the Online Mendelian Inheritance in Man (OMIM) database and annotated using the Human Phenotype Ontology (HPO) to create phenotype profiles for thousands of Mendelian diseases. Each individual in a cohort is assigned to a score based on the presence or absence of matching features for the target Mendelian disease. The HPO provides a standardized vocabulary of characteristic abnormalities encountered in human disease. HPO terms can then be mapped to consolidated billing codes (phecodes), individual codes in ICD9 or ICD10, or other information extractable from the EHR, establishing well-coded disease definitions. By then assessing the presence of these features within the record of a patient of interest, one can apply a predictive lens. Specifically, the PheRS for a given Mendelian disease is defined as the sum of clinical features observed in a given subject weighted by the log inverse prevalence of the feature—essentially a disease likelihood based on tractable canonical disease symptom overlap. After initially being used to assess the pathogenicity of rare genetic variants, PheRSs were refined to serve as a scalable approach to identifying undiagnosed disease and assessing gene expression (9, 10, 91, 122).

EHRs have also been used to interpret clinical genetic sequences more efficiently. Interpreting clinical genetic data often requires manual chart abstraction to help prioritize and interpret genetic variants. Tools like ClinPhen have been designed to automate this process, using natural language processing techniques to extract clinical concepts relevant to Mendelian disease diagnosis and map them to the HPO. Clark et al. (16) described an automated pipeline that extracts features from an EHR and pairs them with whole-exome sequencing results.

### Applying Discovery Research Methods to Translational Medicine

Utilizing genomic data in a clinical setting can be associated with many barriers, including operational issues, physician comprehension of the results and attitudes toward genetic data, determining how to effectively utilize results, clinical decision support, integration of the result data into the EHR itself, and concerns about associated costs (29, 53). As an example, Vanderbilt University Medical Center developed a research program in 2010 aimed at determining the effectiveness of preemptive pharmacogenomic testing of high-risk patient populations to decrease medication-related adverse events. This program, called the Pharmacogenomic Resource for Enhanced Decisions in Care and Treatment (PREDICT) (81), combined genomic testing, integration of the results into the EHR, and associated clinical decision support for physicians. Physician attitudes were studied, and while the majority agreed that immediate notification of significant drug–genome interaction was beneficial, there were divisions regarding the responsibility of the physician, which physicians should be notified, and whether patients should be notified directly (79). Nationwide surveys supported the findings, suggesting that physicians did not feel prepared regarding pharmacogenomic testing (99).

As pharmacogenomic testing has become more common, physicians have become more familiar with resources such as the Pharmacogenomics Knowledge Base (PharmGKB) (112), the Pharmacogenomics Research Network, and the Clinical Pharmacogenetics Implementation Consortium guidelines (82). While acceptance and utilization of pharmacogenomics are still challenges (37, 40), recent studies have shown that education and even having physicians undergo personal genomic testing can greatly alter attitudes and understanding (59). Examples from the pharmacogenomics field can inform barriers to genomic medicine in general. Clinical education, understanding, and support are key for the successful integration of genomics into a healthcare setting.

Moving from discovery research to translational medicine and ultimately informing changes in patient care has been the focus of billions of dollars of research in countries across the whole world for the last decade (101). Many of these countries nationally fund networks and research programs whose main goals are to overcome barriers to implementing genomic medicine in clinical practices and determine best practices and lessons for translational medicine as a whole. Lessons from these networks inform the integration of genomics into healthcare research. Two networks funded by the National Human Genome Research Institute have focused on large-scale EHR research and integrating genomic results into translational research and clinical practice: the eMERGE network and the Implementing Genomics in Practice (IGNITE) network.

The eMERGE network moved from discovery research focused on GWASs (27, 85), PheWASs (23, 24, 107), and e-phenotyping (48, 92) in its earlier phases to returning and integrating actionable genomic variants (31, 32). The network is currently investigating how genomic and polygenic risk factors integrate and associate with development of common complex diseases. It led the field in the reuse of EHR data for secondary research (20, 41, 55, 68, 78), in addition to developing methods for the integration of genomic results and assessment of clinical uptake and utilization of genomics over the last several years (6, 21, 36, 46, 88, 113). Lessons from the network (32) include up-front data requirements; rapid sharing of data across EHR systems by using standardized common data models and collection with local expertise; strong centralized communication, policies, and project management; consistency in methods; harmonization of data flow and integration utilizing Health Level 7 International (HL7) and Fast Healthcare Interoperability Resources (FHIR) standards when possible; and a specific study design with the identification of attainable short- and long-term goals for downstream analysis of clinical utilization and uptake across sites for future downstream analyses (31).

The IGNITE network focuses on accelerating genomic medicine utilization by developing methods for incorporating genomics into clinical care across diverse settings. With an emphasis on implementation science, the lessons from the IGNITE network highlight the importance of having transdisciplinary teams to ensure appropriate expertise during implementation, understanding the educational needs of clinic providers and staff and having appropriate tools to address these needs, carrying out patient education and engagement, and (as mentioned above for the eMERGE network) having specific study designs for the outcomes of interest and strong IT support and data flow standards (39, 98). The IGNITE network also identified that increasing the priority at the institution of integrating genomics within the health system EHRs by utilizing data warehouses can assist with overcoming integration challenges (98). The IGNITE network’s Clinical Informatics Working Group recently published a framework data flow for germline genomic result generation and integration into an institution’s EHRs from both external and internal vendors (25). The framework, validated through a survey at both IGNITE and eMERGE institutions, highlights the importance of the automation and standardization of genomic information and reporting across the pipeline to enhance utility and streamlined integration, since the knowledge bases associated with genomic medicine are constantly evolving (25). These lessons are applicable not only to other consortia but also to the transition from discovery to translational research.

### The Challenges of Clinical Utility and Implementation

**Figure 4:**
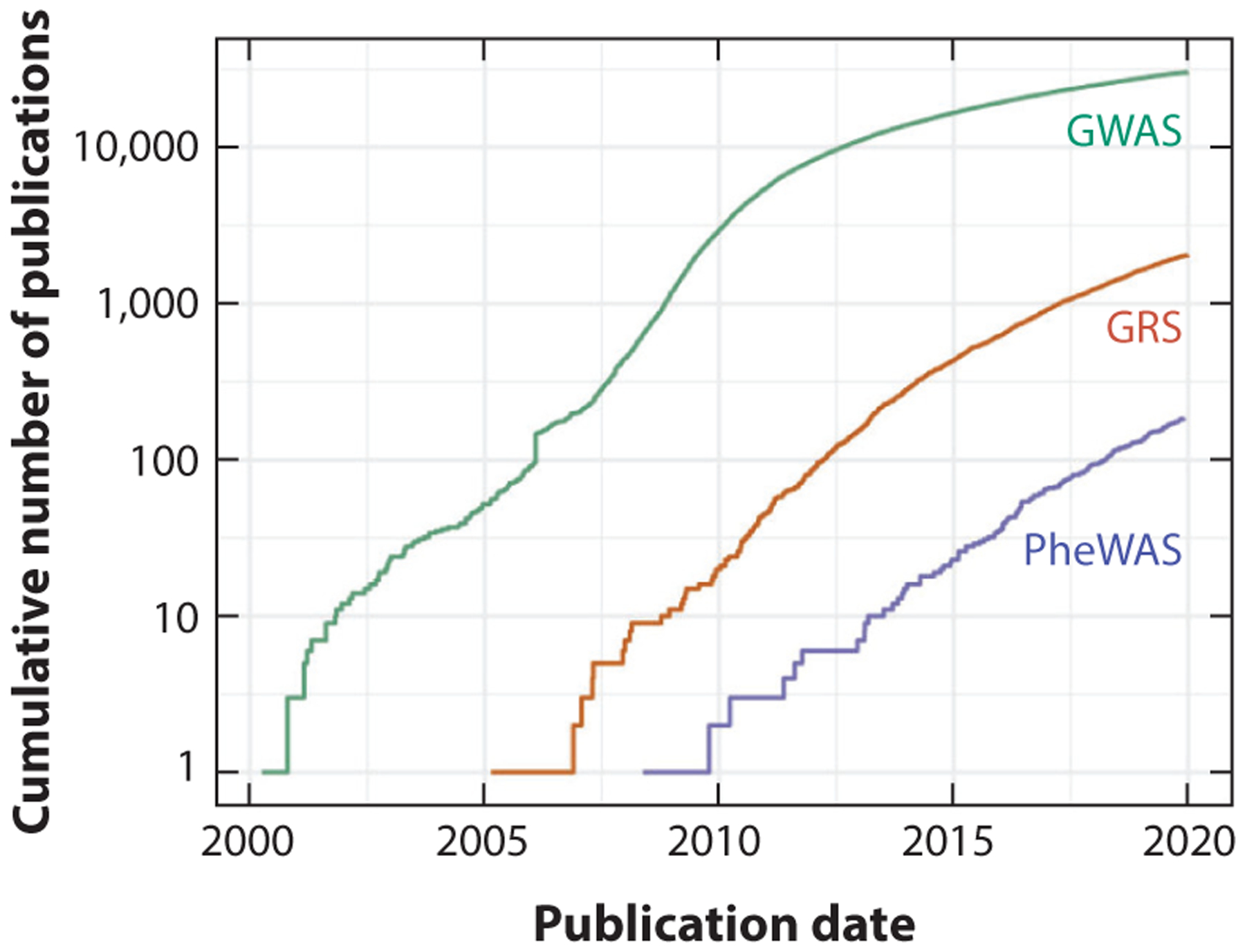
Cumulative number of publications that included terms for common large-scale analytic methods (GWAS, genome-wide association study; PheWAS, phenome-wide association study, phenome wide; GRS, genomic risk score, genetic risk score, polygenic risk score) in the title, abstract, or MeSH term. Enabling methods such as GWAS and PheWAS in combination with the availability of large-scale EHR data laid the foundation for translational research such as PRSs and GRSs. Abbreviations: EHR, electronic health record; GRS, genomic risk score; GWAS, genome-wide association study; MeSH, Medical Subject Headings; PheWAS, phenome-wide association study; PRS, polygenic risk score.

The growth of large-scale genomic and phenomic analyses and the resulting genomic risk scores (GRSs) have greatly increased the opportunities for translating genomics to clinical practice. Publications referencing GWASs, PheWASs, and GRSs were introduced between 2000 and 2010 but exponentially increased early in the last decade and have continued to grow (Figure 4). The majority of these publications describe applications of these three methods to new clinical domains. However, there have been few published studies of translation, implementation, or clinical utility. Such studies have been initiated and include the latest phase of eMERGE, which began in mid-2020 and is investigating genomic risk assessment and management. During this phase, the network is focusing on returning an integrated risk to participants that incorporates PRSs, family history, clinical risk factors, and monogenic risks.

In addition, clinical trials to study early-phase commercial products featuring PRSs are recruiting participants. Both the commercial and academic environment have recognized the challenge of using PRSs in practice; many are based on GWAS data that do not include diverse ethnic representation and consequently perform poorly for people with non-European ancestry (28). The actionability of PRSs for diseases with a long latency (such as many cancers and cardiovascular disease) is also not established for all age ranges, and outcomes are difficult to study given the need for long follow-up. Given this state, most experts are cautious regarding the utility of applying PRSs to clinical care. The National Comprehensive Cancer Network guidelines specifically referenced PRSs in a 2020 update to discourage clinical use outside of clinical trials or until their interpretation and therapeutic implications could be clarified (22). The ability of PheRSs to identify patients with genomic syndromes is also not yet established, in part because the technique requires very large populations with detailed EHRs to accrue a sufficient number of patients with a latent or unrecognized genomic syndrome.

Barriers to the adoption of genomic medicine, including complex interventions such as PRSs, have been identified at multiple institutions. Chief among these are provider uptake, education, and willingness to integrate into clinical care (79, 99). As longitudinal EHR databases become more accessible across diverse populations and tied to banked genetic data, the field of clinical genomics will rapidly expand. Networks that aim to increase diversity in recruited population cohorts, such as the *All of Us* Research Program and eMERGE, will provide researchers with the diverse genetic samples that have previously been lacking from studies of large cohorts. Studies demonstrating the feasibility and clinical utility of these new techniques in diverse populations are critical for widespread adoption. Despite these reservations, it is likely that both PRS and PheRS interventions will follow the path of more established genomic medicine inventions, such as pharmacogenomics and the diagnosis of unknown diseases, which gradually gained acceptance in the clinic as clinical trials and implementation cohort studies were completed (64, 86, 114). Clinical trials have begun on PRSs over the last few years (72), and more trials are expected as the PRS model is vetted across multiple populations. Successful completion of current and future consortia will be needed to formally test clinical use. Since genomic medicine is a relatively young field, common approaches to outcome assessment for polygenic risk will need to be reconciled across studies and established, similarly to the approach taken for monogenic disease (80).

## CONCLUSIONS

High-throughput and large-scale methods for associating genomic and phenomic data have accelerated the discovery of large sets of genetic markers with potential prognostic clinical value. The methods are increasingly dependent on the availability of comprehensive and longitudinal EHR data using structured data linked to sequence data on very large populations. These innovations have fueled the development of new risk stratification and predictive tools that have proven value for discovery, particularly the ability to characterize rare variants and pleiotropy, and have promising but unproven clinical value. For clinical use, there is a need to define the actionability of the predictive information, perform additional validation across ethnicities, and perform outcome-based studies. These advances in translational genomic medicine are founded on the collaborative nature of cross-institutional and global data sharing made possible by the advances in EHR utilization over the past few decades.

## Figures and Tables

**Table 1:** Commonly utilized data elements and tools for extracting phenotypes from EHRs for phenome-wide research

Data element	Description	Utility for phenome science
Claims data	Billing claims data used for diagnosis and procedures; examples include ICD, phecodes (derived from ICD), and CPT	Structured data to extrapolate patient diagnoses, symptoms, findings, and procedures
Demographics	Age, sex/gender, race, ethnicity, date of birth, date of death	Covariate adjustments, cohort definition, structured data
Indexed concepts from clinical narratives	Terms may be mapped to SNOMED-CT and the HPO	Standardizing phenotype concepts to index and merge narrative text
Semistructured documents	Problem lists, family history, flow sheets, radiology, pathology, procedures, cytology reports	Natural language processing for complex e-phenotyping
Encounters	Admission-discharge-transfer, provider and clinic assignments	Severity stratification, healthcare utilization
Laboratory	Laboratory name, value, unit, date; standardized by LOINC in some EHRs	Criteria for detecting diagnoses, cohort definitions, covariate adjustments
Medications	Medication name, dosing, frequency, route, duration, form, strength standardized to RxNorm standard	Criteria for detecting exposures, cohort definitions, covariate adjustments
Tumor (cancer) registry	Organization (e.g., North American Association of Central Cancer Registries) for cancer registry data across public and private organizations for standardization	Cancer-related e-phenotyping
Vital signs	Blood pressure, BMI, height, weight, temperature	Covariate adjustments, structured data

Abbreviations: BMI, body mass index; CPT, Current Procedural Terminology; EHR, electronic health record; e-phenotyping, electronic phenotyping; HPO, Human Phenotype Ontology; ICD, International Classification of Diseases; LOINC, Logical Observation Identifiers Names and Codes; SNOMED-CT, Systematized Nomenclature of Medicine–Clinical Terms.

## CREATING A PHENOTYPE RISK SCORE

A PheRS is a numeric value assigned to an individual based on the number of features they share with the clinical description of a disease.

### Calculating the Phenotype Risk Score

PheRSs are calculated by summing up the weights of each feature present in the EHR (Figure 3a). The weights are defined as the −log_10_ of the prevalence of the phenotype in a large cohort. Different scores can be calculated for different sets of phenotypes. The phenotype sets can be defined based on the clinical manifestations of a particular disease or created de novo to describe a particular patient. The score is intended to reflect the degree of similarity between a patient and the feature set.

### Phenotype Risk Scores for a Mendelian Disease

OMIM provides clinical descriptions for thousands of Mendelian diseases. These descriptions have been annotated with HPO terms, and a map has been created between HPO terms and phecodes; thus, every disease in OMIM can be described as a set of phecodes. Some HPO terms are mapped to phecodes that match exactly, while others are mapped to broader phecodes. Figure 3b shows an abbreviated version of OMIM’s clinical description for cystic fibrosis. When PheRSs are applied to a cohort, they can distinguish between cases and controls for a particular disease without relying on the disease label itself. PheRSs have been used to identify pathogenic variants in HER-linked biobanks.

### Finding Undiagnosed Patients

Beyond using PheRSs to study rare genetic variants, preliminary work suggests that they may help identify undiagnosed patients. For example, in Figure 3c, a patient diagnosed with cystic fibrosis late in life had a PheRS in the 99th percentile prior to diagnosis.

## Abbreviations

EHR: electronic health record
PheWAS: phenome-wide association study
GWAS: genome-wide association study
PheRS: phenotype risk score
PRS: polygenic risk score
